# Do preterm-born children and adolescents have greater need for dental care as compared to full term-born controls?

**DOI:** 10.1186/s12903-022-02504-8

**Published:** 2022-11-09

**Authors:** António Vicente, Lubna Alward, Anna-Paulina Wiedel, Magnus Becker, Xie-Qi Shi, Kristina Hellén-Halme, Susanne Brogårdh-Roth

**Affiliations:** 1 Department of Oral and Maxillofacial Radiology, Faculty of Odontology, Malmö University, SE-205 06 Malmö, Sweden; 2 Department of Paediatric Dentistry, Faculty of Odontology, Malmö University, Malmö, Sweden; 3 Department of Oral and Maxillofacial Surgery, Skåne University Hospital, Malmö, Sweden; 4 Department of Plastic and Reconstructive Surgery, Department of Clinical Sciences in Malmö, Skåne University Hospital, Lund University, Malmö, Sweden; 5 Section of Oral and Maxillofacial Radiology, Department of Clinical Dentistry, University of Bergen, Bergen, Norway

**Keywords:** Children, Dental radiography, Intraoral imaging, Preterm birth

## Abstract

**Background:**

Preterm birth has been shown to cause various long-term health issues. Children who were born preterm have also been observed to have more dental behaviour management problems (DBMP) during dental examinations and treatment than children born full term. It is known that dental radiographic examinations can be uncomfortable and cause anxiety in paediatric patients. Thus, our aims were to retrospectively compare dental care related examinations and treatments carried out in three different age intervals (3–6 years, 7–12 years, and 13–19 years) among preterm- and full-term born children and adolescents.

**Methods:**

The present study included 311 patient files: 122 very preterm–born and 33 extremely preterm–born children and adolescents (< 32 gestational weeks). A matched control group of 156 full term–born children and adolescents (≥ 37 gestational weeks) was analysed for comparison. Various factors, including DBMP, missed appointments, dental caries, and radiographic examinations, were retrieved from the dental records for three age intervals: 3–6 years, 7–12 years, and 13–19 years.

**Results:**

Extremely preterm–born children missed significantly more dental appointments and presented with more DBMP during dental examinations and treatment than full term–born children in the 3–6-year age group; the same was observed for the very preterm–born in the 7–12-year age group. No significant differences in DBMP during bitewing and periapical examinations or in number of bitewing, periapical and panoramic radiographs occurred between the groups in any age interval.

**Conclusion:**

Preterm–born children and adolescents may need more flexibility in booking and receive reminders for scheduled visits with the general dental team. Due to the non-significant differences in dental care related oral examinations and treatments, the same dental care service may be applied to the preterm- and full-term born children and adolescents.

## Background

Despite advancements in medicine and technology that have reduced infant and child mortality, preterm birth is worldwide considered to be the principal cause of death in children under the age of 5 years. The effects of a preterm birth, however, may still surface in long-term health issues of various kinds [1]. The most common, long-lasting effects include chronic lung disease, vison and hearing loss, intellectual impairment, and cerebral palsy [1, 2]. Risk of impairment increases with neonatal complications and decreasing gestational age and birth weight [3]. Oral health may also be affected as a result of medical health problems, such as a complicated delivery, infections, brain injury, or feeding difficulties in the neonatal period. In the dental context, this may affect general oral health as well as other oral health-related habits [4], including behavioural difficulties related to dental treatment. Dental behaviour management problems (DBMP) are defined as lack of cooperation and disruptive behaviours that may cause a delay in, or even lack of, treatment [5]. During the preschool and school years, it has been shown that preterm–born patients had more DBMP during dental examinations and treatment than children born full term [6, 7], as well as a higher number of missed appointments during the preschool years [6]. Nevertheless, some studies have shown that preterm-born children did not have a higher risk of developing caries lesions than full term-born children [6, 8–11] while others have shown the opposite [12, 13]. Other reported oral health problems in preterm–born children (especially in extremely preterm– and very preterm–born children) include disturbances in tooth development, such as smaller teeth, delay in tooth maturation, and more enamel defects compared with full term–born children. High frequencies of temporomandibular disorder pain (TMD pain) during adolescence have also been reported, as well as more malocclusions, and a greater subjective need for orthodontic treatment compared with patients born full term [7, 14–17].

In dentistry, radiographic investigations are helpful for diagnosing and treating oral disease [18]. However, all radiographic examinations must be adjusted, justified, and optimised for each patient, as exposure to ionizing radiation always carries some amount of risk [19, 20]. Sweden has, since 2018, national regulations from the Swedish Radiation Safety Authority which states that the dental records have to include this information [21]. Moreover, because developing tissues are more sensitive to ionizing radiation, children have a higher radiation vulnerability than adults. A small head size also increases the risk that sensitive organs like the thyroid gland may inadvertently become exposed to ionizing radiation [22]. Dental radiographic examinations are one of the most frequent radiographic examinations that paediatric patients take [18], often in combination with periodic dental check-ups for caries detection, dental trauma, and control of disorders in tooth development [23]. Bitewing, periapical, panoramic, and cephalometric images are among the most common dental radiographs made [22]. The panoramic radiograph allows for a wider assessment of the jaws but requires good patient cooperation to obtain acceptable image quality [19, 23]. Brogårdh-Roth et al. [6] observed in their study of 3–6-year-old preterm–born children that fewer dental radiographic examinations were done compared to full term–born children. Intraoral radiographic examinations can be demanding because of the smaller size of the anatomic structures and lack of cooperation [24]. Gagging reflexes due to the detector are relatively common in this population [25]. Moreover, dental radiographic examinations before treatment are often associated with higher anxiety levels in paediatric patients [26]. Brogårdh-Roth et al. found in another study that adolescent girls, both preterm– and full term–born, were more likely to report pain during dental radiographic examinations than boys [27].

To our knowledge, no previous studies have investigated overall oral health and treatment needs, including for dental radiograph examinations, in extremely preterm– and very preterm–born individuals during both childhood and adolescence. Thus, our aims were to retrospectively compare dental care related examinations and treatments carried out in three different age intervals (3–6 years, 7–12 years, and 13–19 years) among preterm- and full-term born children and adolescents. Our hypotheses were that there was no difference between the groups of children and adolescents regarding our aims.

### Clinical relevance

The findings of the present investigation will be helpful in the further development of guidelines and strategies for the dental care of various groups of paediatric patients.

## Materials and methods

### Ethics considerations

The present study followed the tenets of the Declaration of Helsinki. The Ethics Committee of the Medical Faculty of Lund University approved all previous studies from which the material for the present study was collected (Dnr [Daybook no.] LU 362-01; Dnr 618/2007) and also the present study (Dnr Etik H15 2013/39). All methods were performed in accordance with the relevant guidelines and regulations.

An informed-consent form was signed by all participants and, if underage (< 18 years of age), their guardians.

### Study design and participants

All patient data for the extremely preterm– and very preterm–born patients and the control group of full term–born patients were retrieved from previous studies by Brogårdh-Roth et al. [6, 27]. In the present study, the term “preterm” describes children born at 32 weeks of gestation or earlier; “extremely preterm”, children born at 23–28 weeks; and “very preterm”, children born at 29–32 weeks [28, 29]. The sample of dental records included all patients born between 23 and 32 weeks of gestation (n = 192; n = 155 eligible)—that is, the extremely preterm– and very preterm–born groups of adolescents (n = 33 and n = 122 eligible)—during 1994–1996 in the southern Sweden recruitment area of the Lund and Malmö University Hospitals and their matched controls (n = 156). Patients were first identified using the Swedish Medical Birth Registry; data on gestational age, birth weight, and number of siblings were retrieved from the Swedish Board of Health and Welfare. The full term–born control group comprised children who were born at 37 or more gestational weeks and which previous studies had matched with the patients in the two preterm groups [6, 27]. Each control patient was identified through dental clinics and matched for age, sex, immigrant background (at least one parent born outside the Nordic countries), dental clinic, and dentist [6].

The present study retrieved 311 dental records between 1997 and 2015. The sample has participated before in studies made in a thesis by Brogårdh-Roth et al. [4]. When those studies were performed, no other studies focusing on behavioural problems were found. Therefore, an estimation of the sample size was difficult to obtain. In this follow-up study, no sample size calculation was made. The material in this study was based on the original study population.

The inclusion criterion was that dental records for ages 3–19 years were available. The exclusion criteria were missing dental records during ages 3–19 years and declining to participate.

### Dental record review

The Swedish Public Dental Service (Folktandvården), private dental clinics, and the Children’s Dental Clinic at the Faculty of Odontology at Malmö University provided dental records for all participants from 3 to 19 years.

One author (AV) evaluated all dental records (n = 311). For each patient, the following information related to the number of dental visits, radiographic examinations, registered dental caries, registered medical care problems, DBMP, registered sedation and referral needs, were analysed (Table 1). Data on caries prevalence (dft, dt, DFT and DT indexes) is nationally collected in Sweden at four different age groups: 3, 6, 12 and 19 years. The records were blinded according to patient identity and study group. The first 10 reviews were carried out under the supervision of an experienced specialist in paediatric dentistry (SB-R). In cases of doubt or disagreement, specialists were consulted until consensus was reached (SB-R, KH-H).

**Table 1:** Dental record data retrieved for ages 3–6, 7–12, and 13–19 years

Information retrieved from dental records
*Dental visits*
Total number of dental care visits
Total number of dental emergency visits
Total number of visits for behaviour shaping (a learning model to prepare the individual for dental treatment)
Missed appointments (no-shows, late cancellations)
*Radiographic examination*
Total number of bitewing radiograph examinations
Total number of periapical radiograph examinations
Total number of panoramic radiograph examinations
Justification (noted, not noted)
Dental caries at ages 3, 6, 12, and 19 years*
Decayed/filled teeth (dft) – primary dentition
Decayed teeth (dt) – primary dentition
Decayed/Filled Teeth (DFT) – permanent dentition
Decayed teeth (DT) – permanent dentition
*Medical health problems#*
Chronic illness (yes, no)
Daily medication (yes, no)
*Dental Behaviour management problems (DBMP)* [5, 6]
During dental examinations and different treatments, e.g. dental fillings, dental extractions and application of intraoral anaesthetics (yes or no)
During bitewing and periapical radiographic examinations (yes or no)
*Sedation during dental treatment*
Benzodiazepines (yes, no)
Nitrous oxide (yes, no)
General anaesthesia (yes, no)
*Need for referral*
To specialist in paediatric dentistry due to BMP (yes or no)
*Experience of orthodontic treatment, for example, fixed appliances, removable appliances, or both fixed and removable appliances (yes, no).*

* Collected data according to Swedish national guidelines *#* Information gathered from Brogårdh-Roth et al. 2009 [30], and defined according to Westbom & Kornfält [31]

Dental record notes on disruptive behaviour that delayed an examination or treatment, or made dental examinations or treatment impossible, were classified as DBMP [5]. One or more examples of disruptive behaviour during the age intervals 3–6, 7–12, and 13–19 years classified a child/adolescent as having DBMP [5]. Information regarding chronic illness was obtained from an interview study with the parents [30], since the dental staff does not always note this information in the records and the following definition for chronic illness, from Westbom and Kornfält [31], was used:

(1) A disorder which was disabling and obviously chronic or incurable, or (2) a disorder of at least three months during a one-year period and interfering with daily life functioning and/or needing treatment or special aids during at least three months, or (3) a disorder requiring hospitalization for at least one month or at least three periods during a one-year period.

### Statistical analysis

All data were recorded and analysed in Microsoft Office 16 EXCEL (Redmond, Washington, United States) and the Statistical Package for the Social Sciences (IBM SPSS Statistics for Windows, version 27.0. Armonk, NY: IBM Corp). Frequency analyses were done and cross-tabulations were analysed. Pearson’s chi-square test, Fisher’s Exact test, the ANOVA, and Tukey’s Honestly Significant Difference (HSD) test were used for analysing whether between-group differences were significant. The significance level was set at *p* ≤ 0.05. The Bonferroni correction was applied to compensate for the tests used in the different age intervals/groups and therefore counteract the multiple comparisons issue. The significance level was therefore multiplied by three when three age intervals were used (3–6 years, 7–12 years, and 13–19 years) and by four when four age groups were used (3 years, 6 years, 12 years, and 19 years).

## Results

**Fig. 1:**
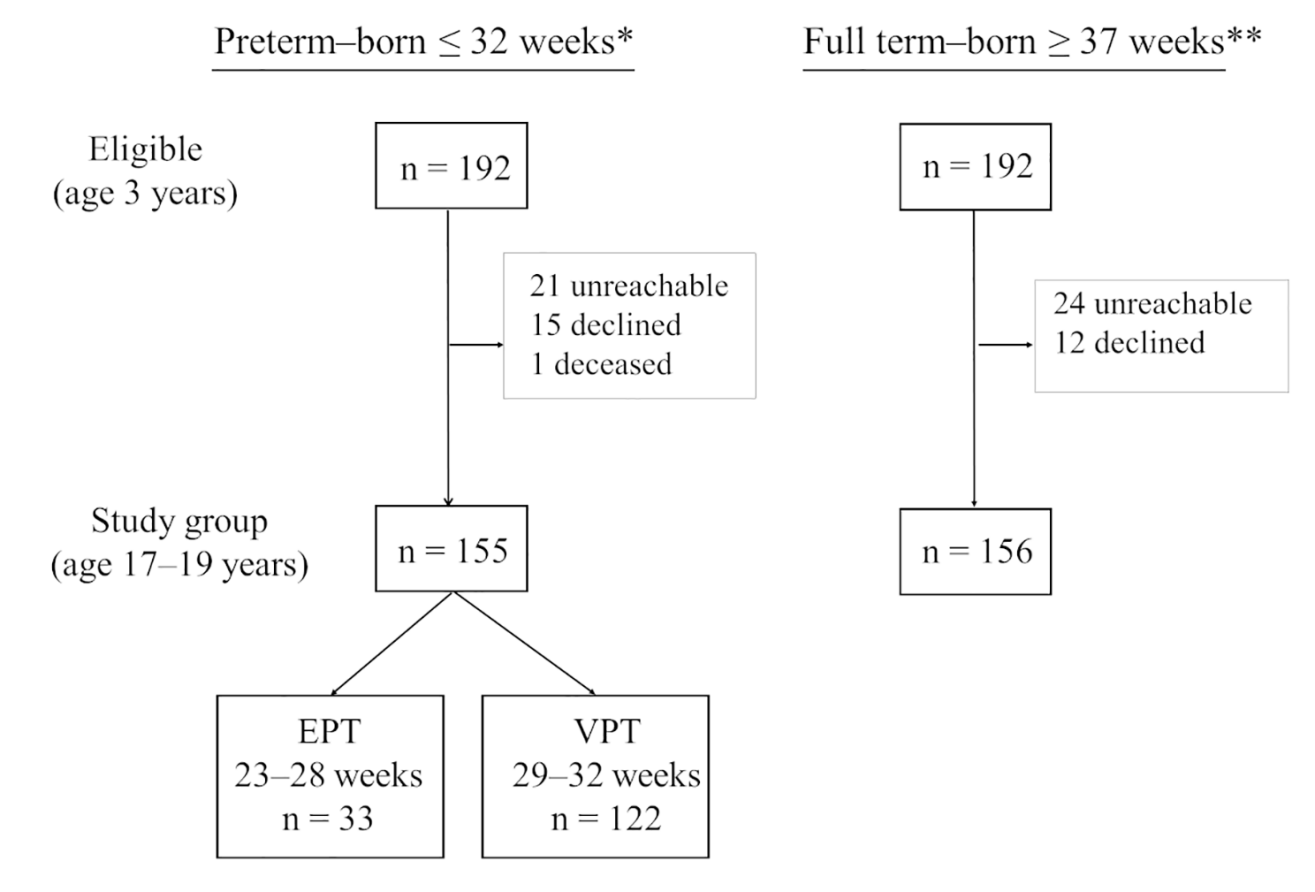
Flowchart of participants eligible at 3 years of age in the present retrospective study Experimental groups: EPT, extremely preterm–born (n = 33); VPT, very preterm–born (n = 122); Control group: FT, full term–born (n = 156) * Identified through the Swedish Medical Birth Register ** Identified through dental clinics Not reachable = No response to mailed written information Declined = Declined participation for reasons unknown Excluded = Dental records were not available throughout for ages 3–19 years

The present study identified 192 eligible preterm–born adolescents and 192 full term–born controls (Fig. 1). Of these, 155 of the preterm–born (80.7%) and 156 of the full term–born (81.3%) adolescents agreed to participate in this study and to allow a review of their dental records from ages 3–19 years. During this period, 83.3% of the participating children and adolescents were regular patients at the Swedish Public Dental Service, 3.5% at private clinics, and 1.6% at the Children’s Dental Clinic at Faculty of Odontology at Malmö University. During this period, the remaining 10.9% had a combination of two caregivers, and 9.0% were patients at the Swedish Public Dental Service or a private clinic. Table 2 presents the birth data and sex distribution of the 311 children and adolescents in the study sample.

**Table 2:** Characteristics of the preterm–born and the matched full term–born children and adolescents

Characteristics	Preterm–born		Full term–born
	**Extremely preterm**	**Very preterm**	
*Sex*			
Male	19 (57.6%)	55 (45.1%)	76 (48.7%)
Female	14 (42.4%)	67 (54.9%)	80 (51.3)
*Twins or triplets*	5 (15.2%)	43 (35.2%)	1 (0.6%)
*Mean gestational age*			
weeks	26.6	30.8	-
range	(24–28)	(29–32)	(≥ 37)
*Mean birth weight*			
grams	936.1	1604.6	3541.5*
range	615–1470	840–2320	2100–4400*

All data retrieved from Brogårdh-Roth et al. 2009 [30]

Extremely preterm–born, n = 33; very preterm–born, n = 122; and full term–born, n = 156

- no data available * n = 128, no data available for n = 28

The two groups of preterm–born children presented with chronic illness more frequently than the full term–born control group. Among the groups, this difference was significant for ages 3–6 years (very preterm and extremely preterm > full term; *p =* 0.004 and *p =* 0.003;) and 13–19 years (extremely preterm > full term; *p =* 0.002).

At 3–6 years, DBMP during dental examination or treatment was found in 17.5% of the children with chronic illness (n = 40) and 19.9% of the healthy children (n = 261). At 7–12 years, it was found in 15.4% of the children with chronic illness (n = 13) and 7.8% of the healthy children (n = 179). At 13–19 years, 7% of the children with chronic illness (n = 43) and 3.1% of the healthy children (n = 229) presented with more DBMP during dental examination and treatment. Even so, a significant relation between the presence of chronic illness and DBMP during dental examination or treatment could not be found for any of the age intervals (3–6 years *p* = 0.719; 7–12 years *p* = 0.296; 13–19 years *p* = 0.198).

### Dental radiographic examinations

During bitewing and periapical examinations, no significant differences in DBMP were found between the three groups for any of the age intervals (Table 3).

**Table 3:** Prevalence of dental behaviour management problems during bitewing and periapical examinations found in the dental records

	Age at radiographic examinations						
	**3–6 years**		**7–12 years**		**13–19 years**		
	*Bitewing radiographs*						
	Total of patients*	n	Total of patients*	n	Total of patients*	n	
**EPT**	14	3 (21.4%)	30	3 (10%)	31	3 (9.7%)	
**VPT**	53	10 (18.9%)	110	7 (6.4%)	113	5 (4.4%)	
**FT**	94	12 (12.8%)	151	12 (7.9%)	150	7 (4.7%)	
*p*-value	1.000		1.000		1.000		
	*Periapical radiographs*						
	Total of patients*	n	Total of patients*	n	Total of patients*	n	
**EPT**	4	0 (0%)	14	1 (7.1%)	12	1 (8.3%)	
**VPT**	24	3 (12.5%)	53	1 (1.9%)	29	0 (0%)	
**FT**	32	2 (6.3%)	76	1 (1.3%)	34	1 (2.9%)	
*p*-value	1.000		0.950		1.000		

Pearson’s chi-square and Fisher’s exact test. Bonferroni correction

Test groups: EPT = extremely preterm–born (n = 33) and VPT = very preterm–born (n = 122); Control group: FT = full term–born (n = 156)

* Total of patients = number of patients in which an attempt to perform a radiographic examination was made

Moreover, no significant differences in mean number of bitewing or periapical examinations occurred between the three groups at any age (Table 4).

**Table 4:** Radiographic examination data from the dental record review

	Age at radiographic examinations								
	**3–6 years**			**7–12 years**			**13–19 years**		
	**Examined patients** **n**	**Exams** **mean** **(SD)**	**Number of exams/pat.**	**Examined patients** **n**	**Exams** **mean** **(SD)**	**Number of exams/pat.**	**Examined patients** **n**	**Exams** **mean** **(SD)**	**Number of exams/pat.**
	*Bitewing radiographs*								
**EPT**	12	0.59 (0.76)	1.58	30	3.68 (1.68)	3.80	30	4.66 (2.18)	4.97
**VPT**	49	0.53 (0.71)	1.29	111	3.09 (1.64)	3.23	116	4.59 (1.81)	4.59
**FT**	100	0.77 (0.78)	1.19	149	3.48 (1.68)	3.50	150	4.83 (1.30)	4.83
Total		0.66			3.35			4.72	
*p*-value		0.093			0.261			1.000	
	*Periapical radiographs*								
**EPT**	4	0.16 (0.45)	0.50	14	0.90 (1.30)	2.00	12	0.91 (2.09)	2.42
**VPT**	100	0.34 (0.86)	1.93	88	1.19 (1.89)	1.57	29	0.82 (1.96)	3.28
**FT**	28	0.28 (0.64)	1.50	74	1.29 (1.93)	2.61	33	0.63 (1.51)	7.06
Total		0.29			1.21			0.73	
*p*-value		1.000			1.000			1.000	

ANOVA and Tukey’s HSD test (Exams mean). Bonferroni correction

Test groups: EPT = extremely preterm–born (n = 33) and VPT = very preterm–born (n = 122); Control group: FT = full term–born (n = 156); SD = standard deviation; pat. = patient

At 3–6 years, 4 panoramic radiographs were taken in the full-term born group. At 7–12 years, 4 panoramic radiographs were taken in the extremely preterm-born group. In the very preterm-born group and the full term-born group, seventeen panoramic radiographs were taken. At 13–19 years, 8 panoramic radiographs were taken in the extremely preterm-born group, twenty-two in the very preterm-born group and eighteen in the full term-born group. No significant differences were found between the three groups at any age (3–6 years: *p =* 0.815; 7–12 years: *p =* 1; 13–19 years: *p =* 0.539).

Justification for a bitewing examination was noted in 1.3% of the dental records at 3–6 years, 3.4% at 7–12 years and 8.9% at 13–19 years. For periapical examinations, justification was noted in 94.8% of the dental records at 3–6 years, 93.7% at 7–12 years and 91.8% at 13–19 years. Justification for panoramic examinations was noted in 75% of the dental records at 3–6 years, 94.3% at 7–12 years and 96.4% at 13–19 years.

### Dental examination and treatment

**Fig. 2:**
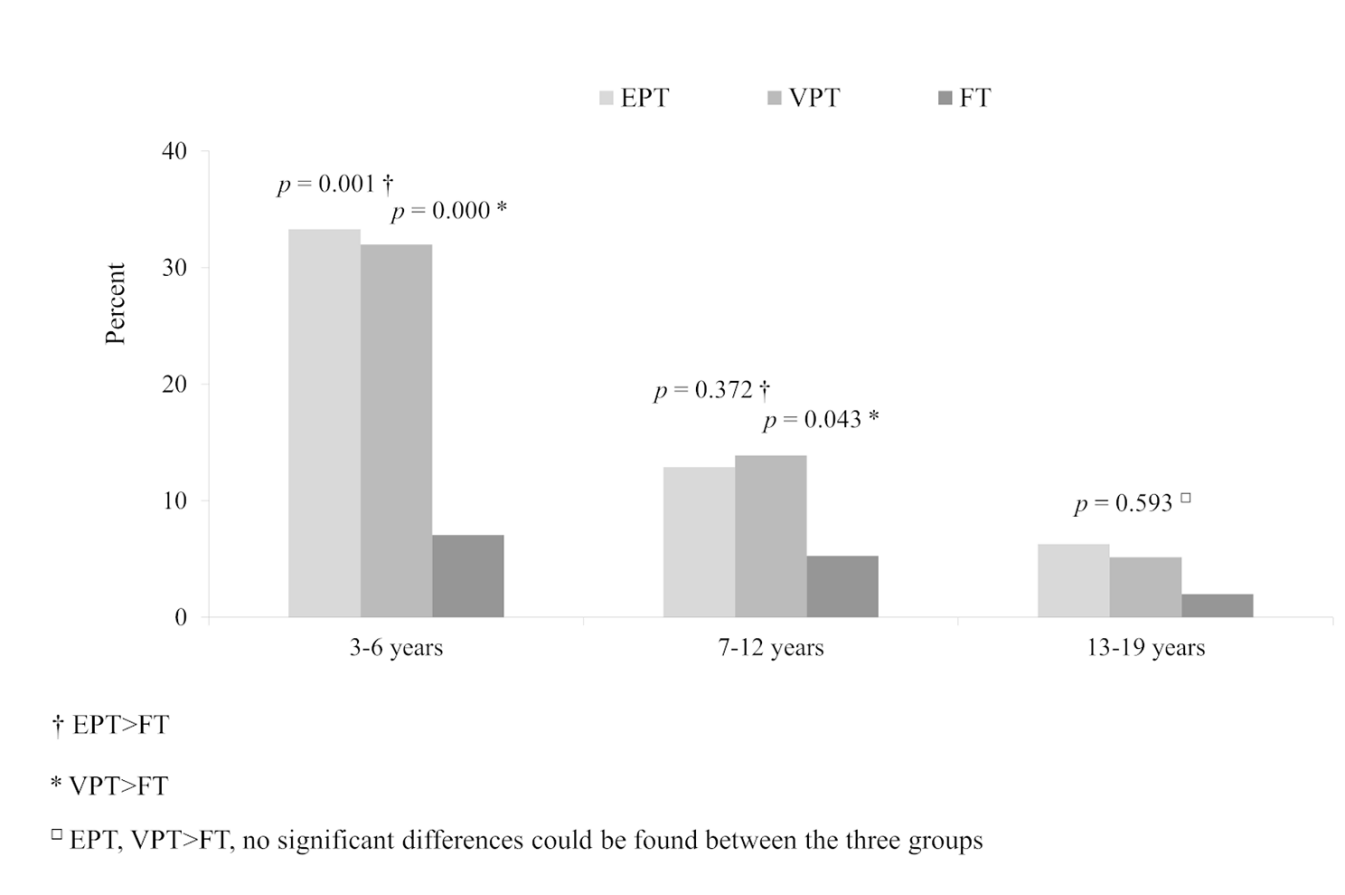
Prevalence of dental behaviour management problems in the extremely preterm-, very preterm– and full term–born study groups Experimental groups: EPT, extremely preterm–born (n = 33); VPT, very preterm–born (n = 122); Control group: FT, full term–born (n = 156) Pearson’s chi-square and Fisher’s exact tests; Bonferroni correction Dental examination and treatment: (e.g., examinations, fillings, extractions, and intraoral anaesthetics)

DBMP occurred more frequently in the two groups of preterm–born children than in the full term–born control group during dental examinations and various treatments (Fig. 2). This was significant at 3–6 years (very preterm and extremely preterm > full term; *p =* 0.000 and *p =* 0.001) and 7–12 years (very preterm > full term; *p =* 0.043), but not at 13–19 years (*p =* 0.593).

### Caries prevalence

**Fig. 3:**
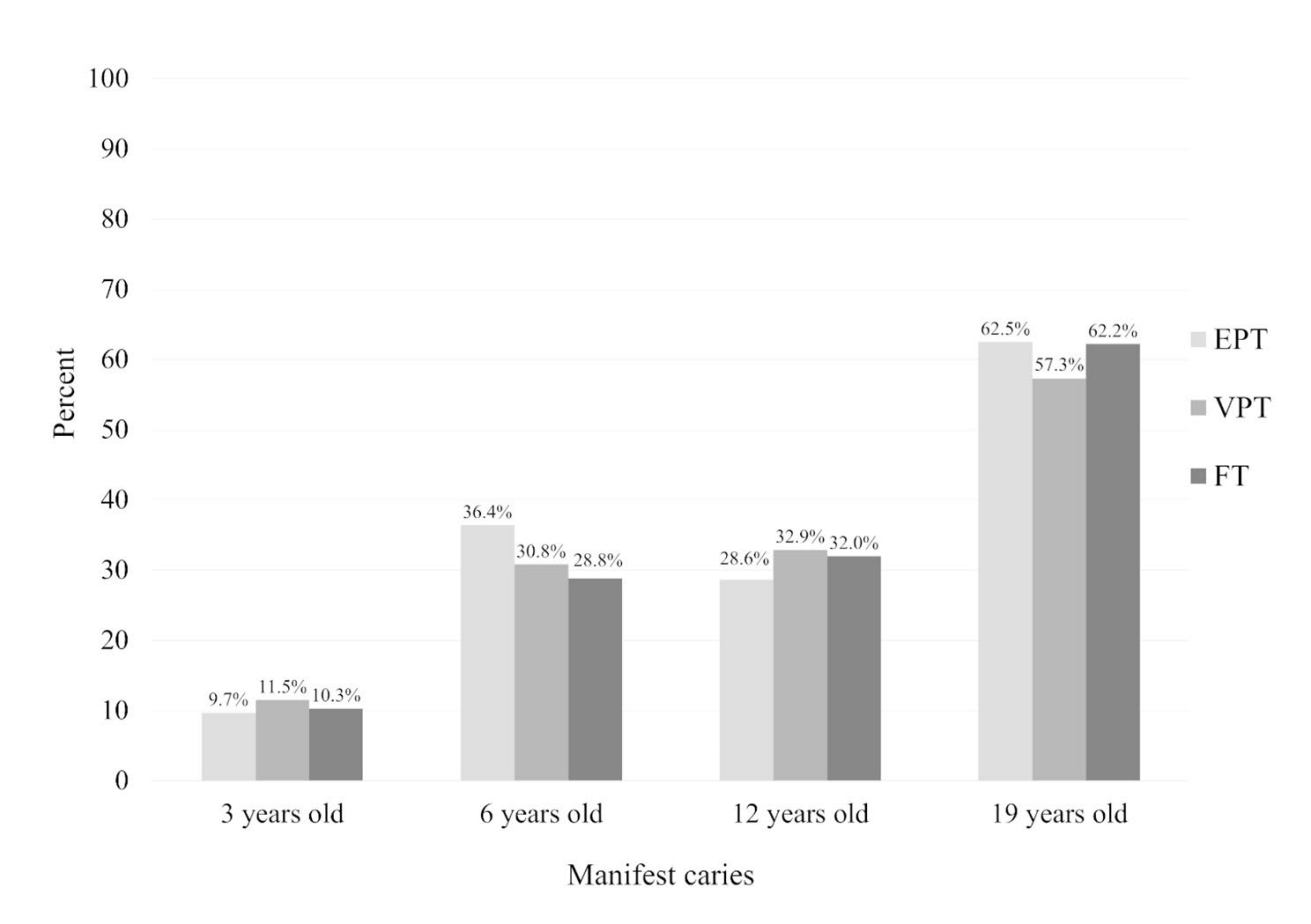
Prevalence of manifest caries in the extremely preterm-, very preterm– and full term–born study groups Experimental groups: EPT, extremely preterm–born (n = 33); VPT, very preterm–born (n = 122); Control group: FT, full term–born (n = 156) Pearson’s chi-square and Fisher’s exact tests; Bonferroni correction No significant differences could be found (*p =* 1.000) between the three groups

**Fig. 4:**
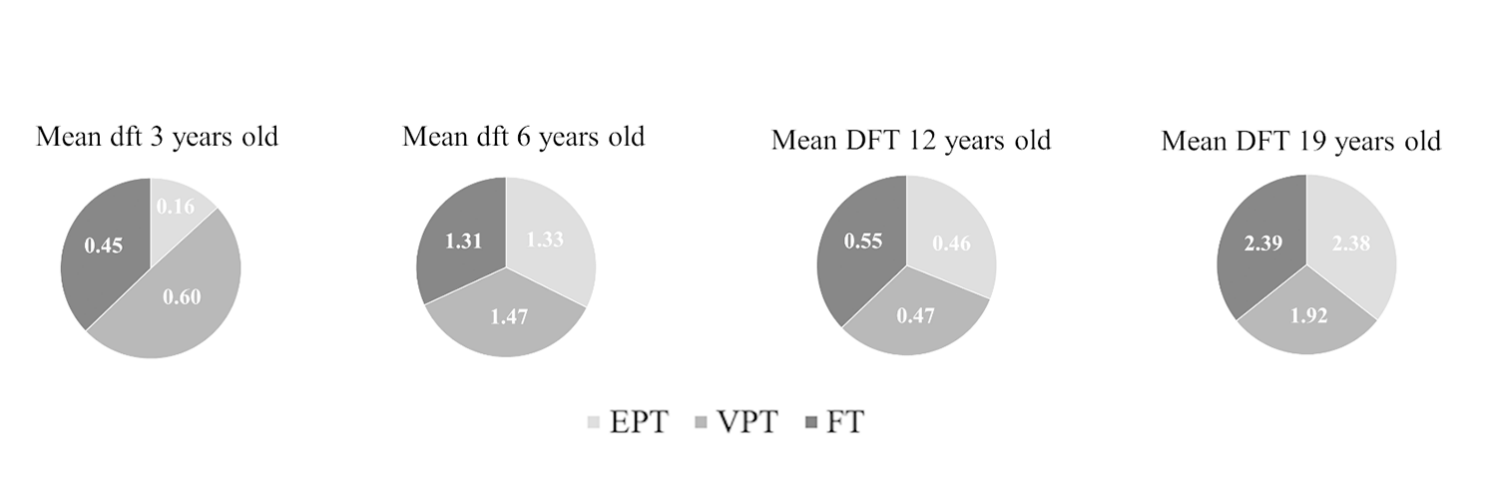
Mean values of decayed/filled teeth (dft/DFT) in the extremely preterm-, very preterm– and full term–born study groups Experimental groups: EPT, extremely preterm–born (n = 33); VPT, very preterm–born (n = 122); Control group: FT, full term–born (n = 156) ANOVA; Bonferroni correction No significant differences could be found (*p =* 1.000) between the three groups

No significant differences in caries prevalence were found between the three groups for any of the age intervals based on registered manifest caries (Fig. 3), and dft/DFT (Fig. 4).

### Other clinical variables

No significant differences could be found between the three groups for sedation, referral to dental paediatric specialist or experience in orthodontic treatment at any age interval (Table 5).

**Table 5:** Sedation during treatment, referral to paediatric dental specialist for DBMP, and orthodontic treatment

	Age interval							
	**3–6 years**			**7–12 years**			**13–19 years**	
	n (total*)	%		n (total*)	%		n (total*)	%
	*Benzodiazepines*							
**EPT**	4 (32)	12.5		0 (31)	0		0 (32)	0
**VPT**	6 (119)	5		4 (116)	3.4		0 (116)	0
**FT**	6 (155)	3.9		0 (153)	0		0 (150)	0
*p*-value	0.383			0.153			-	
	*Nitrous oxide*							
**EPT**	1 (32)	3.1		3 (31)	9.7		0 (32)	0
**VPT**	3 (119)	2.5		8 (117)	6.8		4 (116)	3.4
**FT**	3 (155)	1.9		5 (153)	3.3		0 (150)	0
*p*-value	1.000			0.563			0.162	
	*General anaesthesia*							
**EPT**	0 (32)	0		0 (31)	0		1 (32)	3.1
**VPT**	2 (119)	1.7		0 (116)	0		0 (116)	0
**FT**	1 (155)	0.6		0 (153)	0		1 (150)	0.7
*p*-value	1.000			-			0.611	
	*Referral to a paediatric dental specialist*							
**EPT**	2 (32)	6.3		2 (31)	6.5		1 (32)	3.1
**VPT**	9 (119)	7.6		3 (116)	2.6		0 (116)	0
**FT**	8 (155)	5.2		1 (153)	0.7		1 (150)	0.7
*p*-value	1.000			0.222			0.611	
	*Orthodontic treatment experience*							
**EPT**	0 (32)	0		1 (31)	3.2		13 (32)	40.6
**VPT**	0 (119)	0		13 (116)	11.2		34 (117)	29.1
**FT**	0 (155)	0		11 (153)	7.2		32 (150)	21.3
*p*-value	-			0.968			0.170	

Pearson’s chi-square and Fisher’s exact test. Bonferroni correction

Test groups: EPT = extremely preterm–born (n = 33) and VPT = very preterm–born (n = 122); Control group: FT = full term–born (n = 156)

(total*) = total of patients with available data

### Visits and missed appointments

No significant difference in mean number of dental care visits was found between the control and experimental groups at any age interval (Table 6). Extremely preterm–born missed significantly more dental appointments than full term–born controls at 3–6 years (*p =* 0.002) while very preterm–born children missed significantly more appointments than the controls at 7–12 years (*p =* 0.010). However, no significant differences were found at 13–19 years (*p =* 0.617; Table 6). Neither were any significant differences at any age interval found in mean number of dental emergency visits or visits for behaviour shaping, between the three groups (Table 6).

**Table 6:** Mean numbers, ranges, and standard deviation (SD) of dental care, emergency visits and visits for behaviour shaping, and missed appointments

Group	Mean number, range, and SD												
	**Dental care visits**			**Missed appointments**			**Dental emergency visits**			**Visits for behaviour shaping**			
	**Age interval**			**Age interval**			**Age interval**			**Age interval**			
	**3–6**	**7–12**	**13–19**	**3–6**	**7–12**	**13–19**	**3–6**	**7–12**	**13–19**	**3–6**	**7–12**	**13–19**	
EPT	5.41	10.00	12.25	3.27	3.13	3.22	0.19	0.81	0.63	0.59	0.23	0.00	
Range	1–15	3–27	2–30	0–13	0–14	0–12	0–1	0–4	0–6	0–3	0–3	0	
SD	2.45	5.25	6.00	3.01	3.24	2.85	0.40	1.08	1.16	0.95	0.62	0.00	
VPT	5.44	10.21	9.57	2.17	3.24	3.59	0.36	0.58	0.61	0.50	0.16	0.03	
Range	1–18	1–36	1–32	0–14	0–17	0–24	0–3	0–8	0–7	0–3	0–3	0–3	
SD	3.50	6.63	5.77	2.78	3.84	4.39	0.70	1.07	1.24	0.69	0.53	0.29	
FT	5.65	9.86	9.89	1.54	1.97	2.79	0.36	0.55	0.51	0.44	0.12	0.01	
Range	1–25	1–38	2–26	0–9	0–13	0–19	0–3	0–7	0–5	0–5	0–3	0–1	
SD	2.96	6.45	4.80	1.93	2.47	3.03	0.62	0.97	0.91	0.77	0.38	0.12	
*p-*value	1.000	1.000	0.119	0.002^†^	0.010^‡^	0.617	1.000	1.000	1.000	1.000	1.000	1.000	

Test groups: EPT = extremely preterm–born (n = 33) and VPT = very preterm–born (n = 122); control group: FT = full term–born (n = 156)

ANOVA and Tukey’s HSD test; Bonferroni correction; ^†^ = EPT > FT; ^‡^ = VPT > FT

SD = standard deviation

## Discussion

The present retrospective study of dental records for 3–19-year-old extremely preterm–, very preterm–, and full term–born children and adolescents supported our hypotheses for many of the investigated variables. At younger ages, 3–6 years and 7–12 years, preterm children missed significantly more dental appointments and had more notes on DBMP in their check-up and treatment records than their full term–born counterparts. Overall, the present study has yielded valuable information for future considerations in the dental care of preterm–born adults; one main result is that both preterm– and full term–born children can be offered the same dental care, which is a welcome result from both patient and care provider perspectives.

The overall aim of this study was a longitudinal overview of the dental care given to preterm– and full term–born patients during their growing up years without focusing on the patient perspective. Previous studies have stated that cognitive deficits are often reported in extremely and very preterm–born individuals [27, 32] and that age, is significantly related to behaviour during radiographic examinations because cooperation increases with age [33], which is supported by our findings. Ghanei et al. found that, in general, 19.3% of the time, children experience pain and 17.7% of the time, discomfort during dental radiographic examinations [34]. The findings of the present study show that premature birth does not seem to be an important factor in DBMP during radiographic examinations, although the procedure can be both painful and uncomfortable.

Regarding the documentation of dental radiographs in the dental records, this study found that almost no dental professional documented why bitewing radiographs were necessary since recording justifications for use of ionizing radiation in dental care was not mandatory until 2018 according to Swedish national regulations [21]. Justification of periapical and panoramic radiographs, however, was usually documented. This might be a consequence of the situation during which these dental radiographs were taken or that they served as an answer to a specific clinical problem.

Our study found no significant differences in mean numbers of bitewing, periapical or panoramic radiographs between preterm–born and full term–born children at any age interval.

In our study, extremely preterm–born children was the group that missed most appointments at ages 3–6 years while very preterm–born children missed most appointments at ages 7–12 years. One possible explanation of this finding could be that preterm–born children have usually more medical appointments than full term–born children, and therefore, may feel that a dental appointment is less important. By ages 13–19 years, however, the difference had disappeared, probably because the need for medical care is less as the adolescent matures.

From 3 to 6 years of age, both the very preterm– and extremely preterm–born presented with more DBMP during dental examinations and various treatments compared to the full term–born controls; furthermore, during ages 7–12 years, the very preterm–born patients also presented with more DBMP than the controls. Reports of preterm–born children having more behavioural and emotional problems than full term–born, as Johnson and Marlow have observed [32], may help explain this finding. This difference disappeared at 13–19 years, which may be due to the adolescent phase being a time of greater maturity and, thus, the preterm–born adolescent being able to adjust to dental care situations better; several studies on preterm–born adolescents have reported similar observations [4, 6, 27, 30]. Brogårdh-Roth et al. found that dental fear and anxiety in the preterm–born adolescents were comparable to that found in the full term–born control group [27]. We found no significant difference in caries prevalence between preterm– and full term–born children, as showed by many others [6, 8–11]. However, other studies showed the contrary [12, 13]. It is important to consider though, that the dental caries indexes used in this study, were calculated in Sweden without evaluating missing teeth. This because, missing teeth in children due to dental caries are very limited in Sweden.

Klaassen et al. showed that the most common reason for referral to a paediatric dental specialist was uncooperative behaviour [35]. Our study found no significant differences between preterm– and full term–born children in number of referrals to paediatric dental specialists, which indicates that although preterm–born children may present with more DBMP during dental examination and treatment, general-practicing dentists are still able to manage these patients. The lack of a significant difference in mean number of visits for behaviour shaping between our experimental and control groups supports this.

In line with the study of Brogårdh-Roth et al., which found that preterm–born children are not more exposed to traumatic dental injuries than full term–born children, we found no difference in mean number of dental emergency visits between our experimental and control groups [36]. We also found no differences in orthodontic treatment experience, although Paulsson et al. have reported a higher treatment need in preterm–born children than full term–born children [37]. Nonetheless, Paulsson et al. showed in another study that extremely preterm–born children can experience delayed tooth development, which the general practicing dentist should note [38].

### Study limitations

Deciphering handwritten dental records can be challenging. Regarding DBMP, registration in dental records may vary depending on how and when the records are written, whether at the end of the day, or during the patient visit, as well as which dental care professional who writes them. Further, results of this study should be interpreted with caution, giving the lack of previous data, missing values in the dental records and limited sample size, particularly concerning the extremely preterm–born group, which only had 33 participants; the group size, however, was in line with official Swedish statistics concerning prevalence of birth between 23 and 28 weeks of gestation [39]. Even so, data from this study provides important information for future research in this area.

### Study strengths

Our study followed the same groups of preterm– and full term–born children and adolescents over a long period (3–19 years of age), which can be considered a strength. Further, the original preterm study population included all adolescents born at ≤ 32 gestational weeks in the catchment area of Malmö and Lund in southern Sweden from 1994 to 1996. Participation was relatively high; thus, the data could be helpful in determining population norms.

Another strength of the study was that one examiner (AV) reviewed all notes from the dental records in collaboration with a specialist in paediatric dentistry (SB-R) and a specialist in Oral and Maxillofacial Radiology (KH-H). Well-defined, standardized methods were used in the dental record review.

## Conclusion

Preterm–born children and adolescents may need more flexibility in booking and receive reminders for scheduled visits with the general dental team. Due to the non-significant differences in dental care related oral examinations and treatments, the same dental care service may be applied to the preterm- and full-term born children and adolescents.

## Data Availability

The datasets used and analysed in the present study are available from the corresponding author upon reasonable request.

## Declarations

An informed-consent form was signed by all participants and, if underage (< 18 years of age), their guardians.

## List of abbreviations

DBMP: Dental behaviour management problems
EPT: Extremely preterm−born (gestational weeks 23−28)
VPT: Very preterm−born (gestational weeks 29−32)
